# Generation of an infectious cDNA clone for NADC30-like PRRSV

**DOI:** 10.3389/fvets.2024.1468981

**Published:** 2024-08-14

**Authors:** Yang-Yang Qiao, Hai-Ming Wang, Hui Lu, Yong-Juan Wang, Wei Zhang, Hao Gu, Xue-Hui Cai, Qin-Se Xu, Zhang-Yan Chen, Yan-Dong Tang

**Affiliations:** ^1^Jiangsu Agri-Animal Husbandry Vocational College, Jiangsu, China; ^2^State Key Laboratory for Animal Disease Control and Prevention, Harbin Veterinary Research Institute of Chinese Academy of Agricultural Sciences, Harbin, China; ^3^Heilongjiang Provincial Research Center for Veterinary Biomedicine, Harbin, China

**Keywords:** NADC30-like, infectious cDNA clone, PRRSV, NADC30, PRRSV-2

## Abstract

The porcine reproductive and respiratory syndrome virus (PRRSV) is a highly significant infectious disease that poses a substantial threat to the global pig industry. In recent years, the NADC30-like strain has gradually emerged as prevalent in China, causing a profound impact on the country’s pig farming industry. Therefore, it is important to conduct an in-depth study on the characteristics and gene functions of the NADC30-like strain. An infectious cDNA clone is an indispensable tool for investigating the functions of viral genes. In this current study, we successfully isolated a NADC30-like strain and constructed its full-length infectious cDNA clone. The utilization of this clone will facilitate our investigation into the viral replication, pathogenesis, and immune response associated with the PRRSV NADC30-like strain.

## Introduction

Porcine reproductive and respiratory syndrome virus (PRRSV) is a highly contagious viral disease that affects pigs worldwide, posing a significant threat to the global pig industry. The PRRSVs, belonging to the family *Arteriviridae*, are typically classified into two distinguished species: PRRSV-1 and PRRSV-2 (1–3). China is the largest country for pig production and has the world’s largest market for pork consumption. PRRSV-2, identified as the epidemic strain in China since its outbreak, has caused significant losses for most farms (4–6). The PRRSV-2 can be classified into nine lineages (Lineage 1–Lineage 9), among which Lineage 1, Lineage 3, Lineage 5, and Lineage 8 have emerged in China since 1996 (7, 8). The prevalent PRRSV-2 strains in China are primarily classified into four lineages: Lineage1 (NADC30-like/NADC34-like), Lineage3 (QYYZ-like), Lineage5 (VR2332-like), and Lineage8 (HP-PRRSV-like/CH-1a-like) (9). Currently, Lineage 1 NADC30-like PRRSV and NADC34-like PRRSV have become the main endemic strains in China (9). These new PRRSV variants pose additional challenges as they may exhibit different characteristics compared to previously known strains.

To better understand the characteristics and gene functions of this NADC30-like strain, it is crucial to conduct an in-depth study. One essential tool for investigating viral gene functions is an infectious cDNA clone, which will allow researchers to manipulate specific genes of the virus’s genome, enabling us to study their effects on viral replication, pathogenesis, and immune response (10–12). In current study, we first isolated a NADC30-like PRRSV. Moreover, we constructed a full-length cDNA of this strain. Understanding the characteristics and gene functions of the NADC30-like strain will provide valuable insights into its pathogenicity, aiding in the development of effective control strategies against PRRSV outbreaks in China and globally.

## Materials and methods

### Cells, virus, reagent, and plasmids

MARC-145 cells were stored in our lab as previous works (10, 11). Immortalized porcine alveolar macrophages (iPAMs) were described as our previous work (13). HeN-L1 strain was isolated in a farm at Henan province. The sequence of HeN-L1 strain was confirmed by amplifying the genome as previously described (14). Briefly, viral RNA was extracted using the QIAamp Viral RNA Mini Kit (QIAGEN) according to the instructions, followed by cDNA synthesis using PrimeScript™ II 1st Strand cDNA Synthesis Kit (TaKaRa). Indicated viral fragments were amplified by Q5 High-Fidelity polymerase (NEB). 5′ and 3′ RACE reactions (Invitrogen) were performed to acquire the terminal untranslated regions. The full-length HeN-L1 genome sequence assembled and subsequently submitted to GenBank (Accession No. PQ062578). Our laboratory stores the pOK12, pcDNA3.1(+), and pUC19 vectors. The pEASY-Blunt3 Cloning Kit, a high-efficiency cloning vector kit, purchased from Beijing Quanshi Jin Biotechnology Co., Ltd. (China). ThermoFisher (United States) provided the restriction endonucleases used in this study. Roche (United States) provides the X-tremeGENE HP DNA Transfection Reagent for transfection. Monoclonal antibodies against PRRSV N protein stored in our lab and FITC-labeled goat anti-mouse IgG antibody purchased from Sigma (United States).

### Sequence analysis

Sequence analysis was described as our previous work (13).

### Assembly of full-length cDNA

The restriction enzyme sites present in both the pOK12 vector sequence and HeN-L1 full genome sequence were analyzed using SnapGene software. Subsequently, the pOK12 vector was first modified as shown in Table 1. The MARC-145 cells were infected with HeN-L1 virus at a multiplicity of infection (MOI) of 0.1. When cytopathic effects (CPE) occurred, the viral culture underwent three cycles of freezing and thawing at −80°C, followed by extraction of total RNA from the supernatant using an RNA extraction kit. Then, using the reverse-transcribed cDNA as a template, we employed specific primers listed in Table 1 to amplify the complete sequence of HeN-L1. The fragments were cloned into modified pOK12 vectors individually. Subsequently, the full-length cDNA was assembled.

**Table 1:** Primers for the construction of HeN-L1 infectious clone.

Primer	Sequence (5′–3′)	Position (nt)
1.1F	GATCGATCCACTAGT *ATTTAAAT* GACATTGATTATTGACTAGTTATTAATAGTAATC	(-666)-(-609)
1.1R	GCCAACACCTATACGTCATAGACTATAGGAATTCC	(-12) -19
1.2F	TATGACGTATAGGTGTTGGCTCTATGCCACG	1–31
1.2R	TTAAACAAAGCTCCGCTCGAGAATGCGCGGGACGTCTTC	3,173–3,194
2F	GTATTTAAATGGCCGCTCGAGAAGGCACGAGATGTTAGCAAAACG	3,188–3,209
2R	CTTATCCGACGCGCCGTTTAAACATTGCTCCTTAGTCAGGCCCTGG	7,274–7,297
3.1F	CTCGAGCGGAGCTTTGTTTAAACTGCTAGCCGCCAGCGGCTTG	7,284–7,307
3.1R	ACCAATGATGAACCTGCTGCGCGACACATTAG	9,652–9,682
3.2F	GCAGCAGGTTCATCATTGGTCCACCCGGTG	9,652–9,682
3.2R	CGCGCCAATAGTTTAGCGGCCGCCTGCCGGGTAAGGC	12,500–12,523
4.1F	GCGTCGGATAAGAATGCGGCCGCTCAGATTTTCAAACCCGGTAG	12,485–12,508
4.1R	CATGGTCAATCACTA *GCGATCGC* TGGGTGAAC (AsisI)	13,358–13,390
4.2F	GTTCACCCA *GCGATCGC* TAGTGATTGACCATG	13,358–13,390
4.2R	AATTTCGGCCGCATGGTTCTCGCCAATTAAATAATAC	14,982–15,019
4.3F	AGAACCATGCGGCCGAAATT(A)_32_GG	14,999-(+35)
4.3R	CCACTAGTCCGA *GGCGCGCC* CCATAGAGCCCACCGCATCCCCAGC	(+322)-(+367)
CMV-F	GC *ATTTAAAT* GACATTGATTATTGACTAG (SmiI)	
CMVR	GGACTCATCAGACGCTTAACAAGCTCTGCTTATATAGACCT	
BGH-F	ACTCGGATGGCTAAGGGAGGGCGCTGTGCCTTCTAGTTGCCAGCCATCTGTTGTTTGC	
BGH-R	A *GGCGCGCC* CCATAGAGCCCACCGCATCC (SgsI)	
MSC-F	AAATGGCCGCTCGAGCGGAGCTTTGTTTAAACGGCGCGTCGGATAAGAATGCGGCCGCTAAACTATTGG	
MCS-R	CGCGCCAATAGTTTAGCGGCCGCATTCTTATCCGACGCGCCGTTTAAACAAAGCTCCGCTCGAGCGGCCATTT	

The underlined words indicated the cleavage site of restriction endonuclease.

### Virus rescue

The MARC-145 cells were seeded into a six-well plate and cultured until reaching a confluent monolayer density of 70–80%. Subsequently, the cells were transfected with pOK-HeN-L1 using X-tremeGENE HP DNA Transfection Reagent, while pOK-MCS empty vector used as a control. Once CPE were observed, the viral particles were collected for subsequent experiments as our recent work (10).

### Biomarker detection

The viral RNA of rescued HeN-L1 was extracted, followed by reverse transcription into cDNA. Subsequently, PCR amplification was conducted, and the resulting PCR products were subjected to DNA sequencing.

### Immunofluorescence assay and Western blot

The rescued HeN-L1 was inoculated into MARC-145 cells, followed by IFA or Western blot analysis 48 h post-infection as our previous work (15–17). Additionally, a negative control without virus inoculation was included.

## Results and discussion

### HeN-L1 isolation and sequence analysis

**Figure 1:**
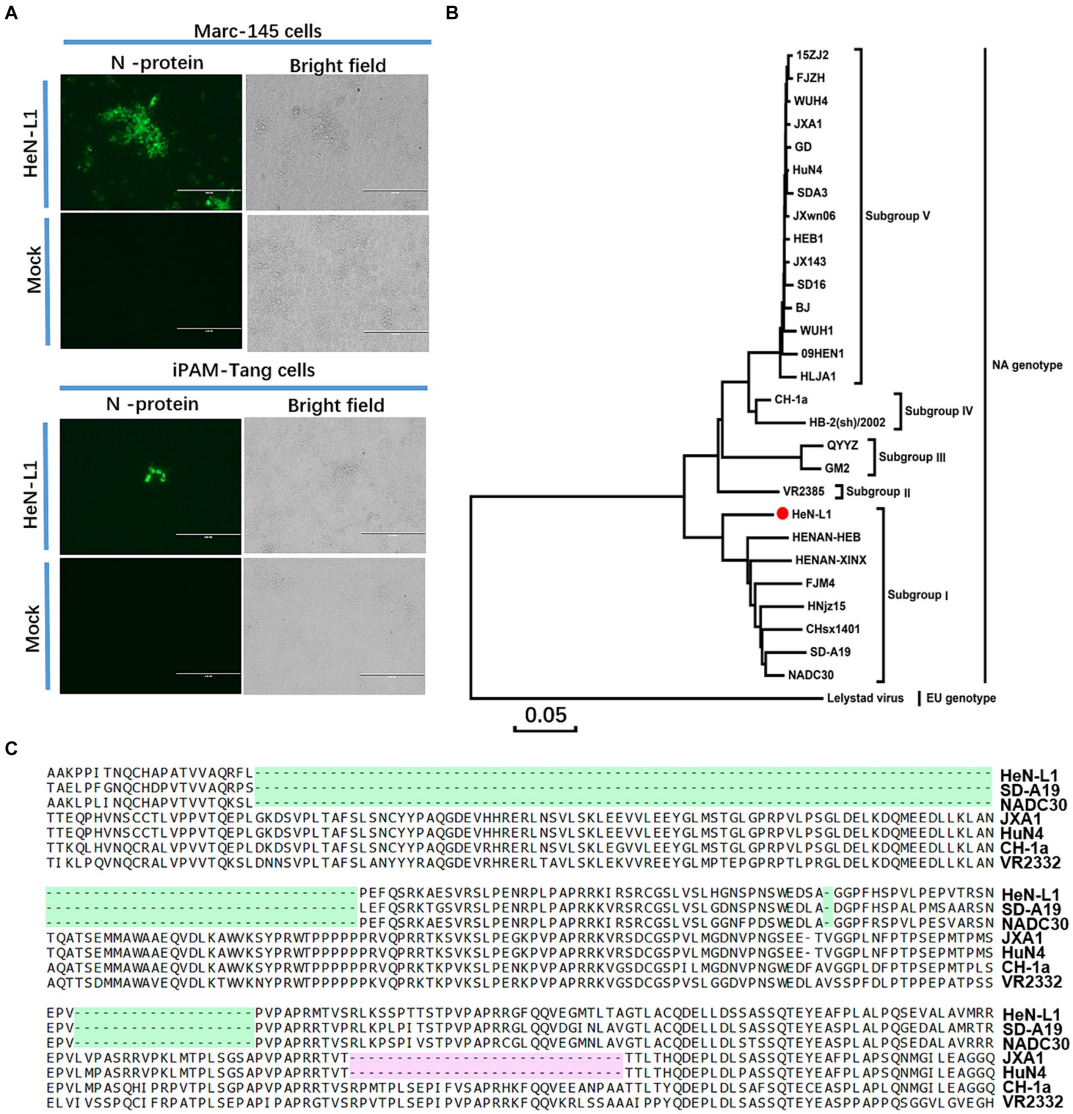
HeN-L1 isolation and sequence analysis. **(A)** MARC-145 cells and iPAM-Tang cells were infected with HeN-L1 strain, respectively. Mock infection as a control. **(B)** Phylogenies of HeN-L1 strain. **(C)** Amino acids alignment for nsp2.

**Figure 2:**
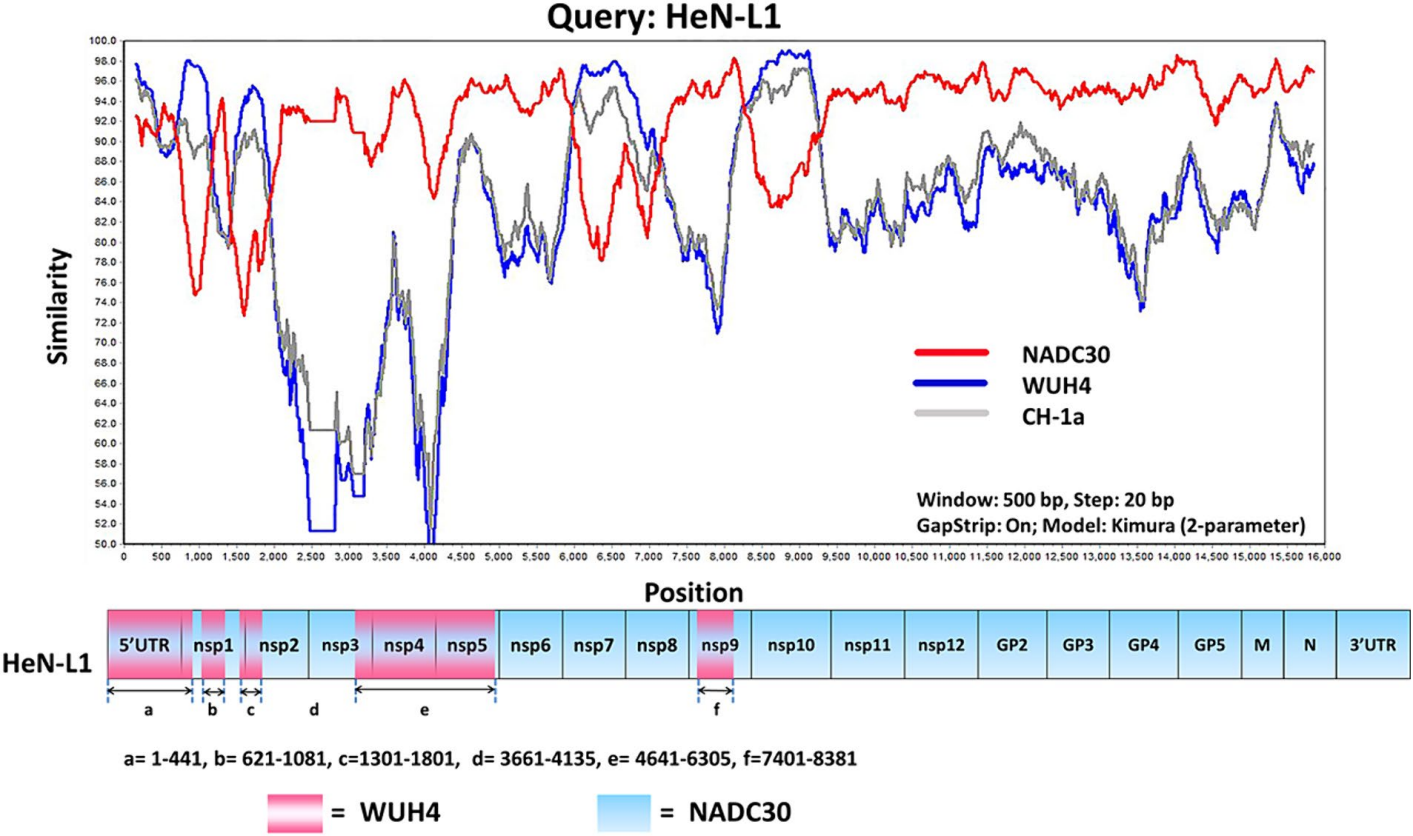
The HeN-L1 strain was subjected to genome recombination analysis using Simplot 3.5.1 software. The complete genome of HeN-L1 was utilized as the query sequence, and the recombination locations are presented at the bottom.

The HeN-L1 strain of NADC30-like PRRSV was isolated from an aborted fetus in Henan province, China. This virus was capable of infecting MARC-145 cells and iPAM-Tang cells (Figure 1A). The full-length genome sequence of HeN-L1 was determined to be 15,017 bp [excluding the poly (A) tail] through DNA sequencing. Phylogenetic analysis revealed that the HeN-L1 strain clustered with other NADC30-like PRRSV isolates such as HNjz15, CHsx1401, and SD-A19 (Figure 1B). To further characterize the HeN-L1 strain, its NSP2 sequence was aligned with reference PRRSV strains. Sequence alignment indicated that HeN-L1 exhibited three discontinuous deletions in NSP2: a 111-amino acid deletion from position 322 to 432, a single amino acid deletion at position 483, and a 19-amino acid deletion from position 504 to 522 (Figure 1C). The recombinant analysis revealed that HeN-L1 is a strain resulting from recombination (Figure 2). These deletions are consistent with those observed in SD-A19 and NADC30 strains. Collectively, these findings preliminarily suggest that HeN-L1 represents one of the circulating strains of NADC30-like PRRSVs in China.

### Construction of full-length HeN-L1 cDNA clone

**Figure 3:**
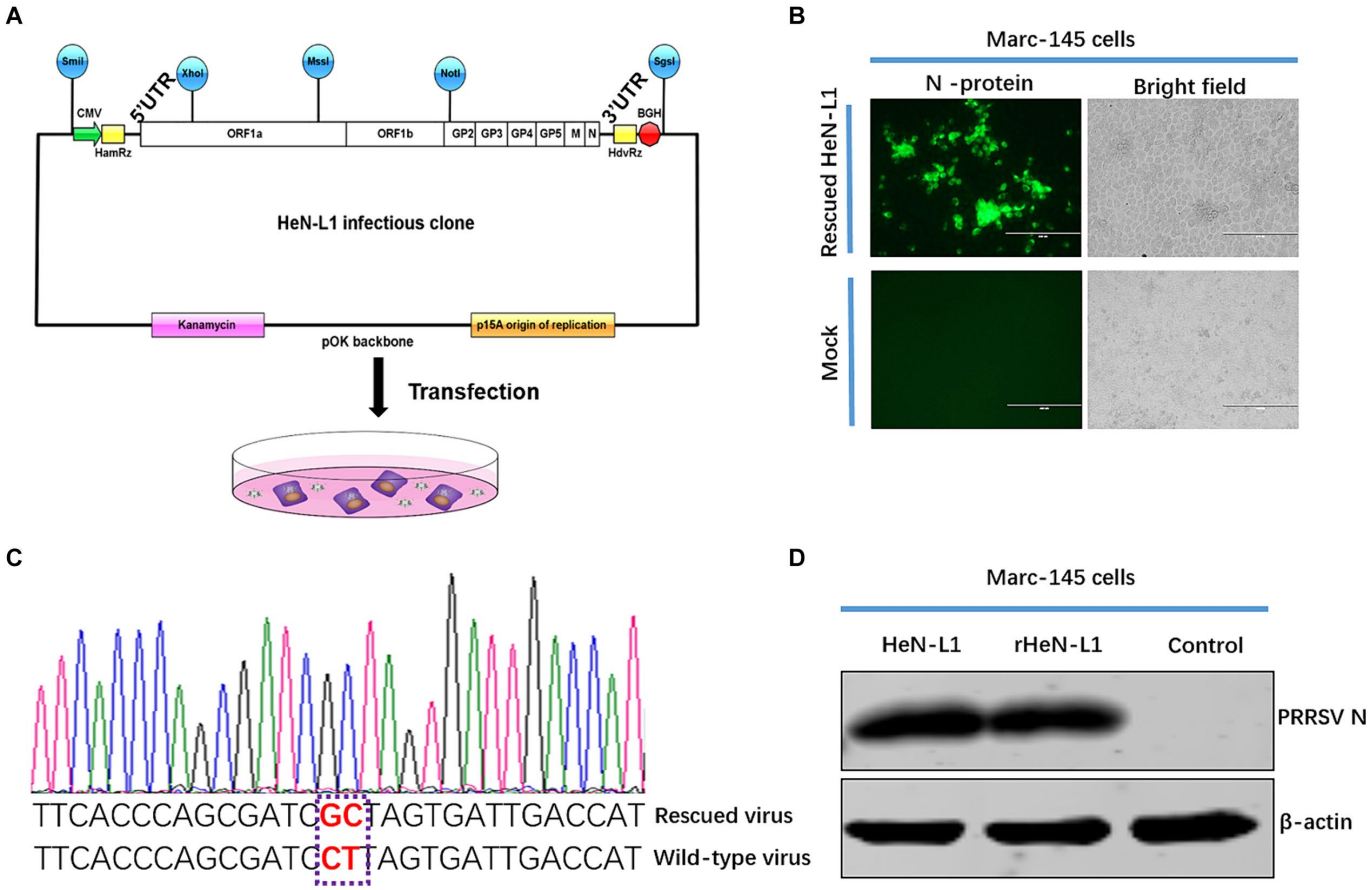
The schematic diagram of the HeN-L1 infectious cDNA clone. **(A)** S protein consists of S1 and S2 domain, and 652 and 661 near the fusion peptide is illustrated. **(B)** MARC-145 cells infected with rescued HeN-L1 strain. Mock infection as a control. **(C)** Sequence analysis of biomarker of rescued HeN-L1 strain. **(D)** The MARC-145 cells were infected with wild-type and rescued virus at a multiplicity of infection (MOI) of 0.1, followed by cell lysis after 24 h for subsequent Western blot analysis.

The infectious clone serves as a crucial platform for investigating the functional aspects of specific viruses and plays a pivotal role in the development of novel vaccines (18–20). A DNA-based approach was employed to generate the infectious clone of HeN-L1 (Figure 3A). The infectious cDNA clone of HeN-L1 strain was generated by inserting full-length genomic cDNA into the low-copy-number vector pOK12 under the control of the eukaryotic RNA polymerase II (Pol II) cytomegalovirus (CMV) promoter. To ensure the release of the authentic 5′ end and 3′end of the viral RNA, the hammerhead ribozyme (HamRz) and hepatitis delta ribozyme (HdvRz) were inserted prior to or after the HeN-L1 genome. Bovine growth hormone polyadenylation signal (BGH) sequence were utilized for efficient transcription termination. In order to differentiate parental virus or clone-derived virus, CT to GC was introduced at 13,281–13,282 nt position of the viral genome. To construct a full-length HeN-L1 cDNA clone, we first infected MARC-145 cells with HeN-L1 at an MOI of 0.01. After 24 h post-infection, the viruses were collected for viral RNA extraction and then reverse transcribed using random primers. The assembly strategy was illustrated in Figure 3A, and we amplified the fragments of HeN-L1 using specific primers listed in Table 1, with the reverse-transcribed cDNA serving as a template.

After assembling the full-length cDNA, we recovered infectious virus by transfecting the viral cDNA clone into MARC-145 cells. The cytopathic effect (CPE) became visible on day 4 post-transfection (data not shown). The rescued virus was further confirmed by re-infecting MARC-145 cells. An indirect immunofluorescence assay against PRRSV N protein revealed a significant number of cells expressing viral N protein on day 3 post-infection (Figure 3B). To ensure that this virus was not contaminated with wild-type virus, we extracted RNAs and amplified the fragment containing a biomarker of the viral genome using RT-PCR. After DNA sequencing, the genetic marker was identified in the rescued virus (Figure 3C). We further confirmed rescued virus by Western blot (Figure 3D).

Overall, the present study successfully isolated a NADC30-like PRRSV and constructed an infectious cDNA clone of this virus. The utilization of this platform will enhance our future investigations aimed at comprehending the characteristics of NADC30-like PRRSV.

## Data availability statement

The datasets presented in this study can be found in online repositories. The names of the repository/repositories and accession number(s) can be found in the article/supplementary material.

## Author contributions

Y-YQ: Data curation, Funding acquisition, Investigation, Writing – original draft. H-MW: Data curation, Formal analysis, Funding acquisition, Investigation, Methodology, Resources, Writing – original draft. HL: Project administration, Resources, Software, Writing – original draft. Y-JW: Methodology, Resources, Software, Validation, Writing – original draft. WZ: Methodology, Software, Validation, Visualization, Writing – original draft. HG: Methodology, Resources, Writing – original draft. X-HC: Conceptualization, Resources, Writing – original draft. Q-SX: Conceptualization, Resources, Supervision, Writing – original draft, Writing – review & editing. Z-YC: Conceptualization, Resources, Supervision, Writing – original draft, Writing – review & editing. Y-DT: Conceptualization, Supervision, Writing – original draft, Writing – review & editing.
